# Association between Internet Use and Locomotive Syndrome, Frailty, and Sarcopenia among Community-Dwelling Older Japanese Adults

**DOI:** 10.3390/nursrep14020105

**Published:** 2024-05-31

**Authors:** Tamaki Hirose, Yohei Sawaya, Masahiro Ishizaka, Naori Hashimoto, Miyoko Watanabe, Masafumi Itokazu, Akira Kubo, Tomohiko Urano

**Affiliations:** 1 Department of Physical Therapy, School of Health Sciences, International University of Health and Welfare, 2600-1 Kitakanemaru, Otawara 324-8501, Tochigi, Japan; n-tamaki@iuhw.ac.jp (T.H.); sawaya@iuhw.ac.jp (Y.S.); ishizaka@iuhw.ac.jp (M.I.); mwatanabe@iuhw.ac.jp (M.W.); itokazu@iuhw.ac.jp (M.I.); 2 Senior Services Division of Otawara, 1-4-1 Honcho, Otawara 324-8641, Tochigi, Japan; hsmt.otawara@gmail.com; 3 Department of Physical Therapy, School of Health Sciences at Odawara, International University of Health and Welfare, 1-2-25 Shiroyama, Odawara 250-8588, Kanagawa, Japan; akubo@iuhw.ac.jp; 4 Department of Geriatric Medicine, School of Medicine, International University of Health and Welfare, 4-3 Kozunomori, Narita 286-8686, Chiba, Japan

**Keywords:** frailty, Internet, Japan, locomotion, sarcopenia

## Abstract

In the lives of those who are the target of community health nursing, it is important to collaborate with individuals and communities to improve their quality of life. Herein, we aimed to determine the association between Internet use among older individuals and locomotive syndrome (LS), frailty, and sarcopenia. In this cross-sectional study conducted between July 2022 and March 2023, we recruited 105 community-dwelling older Japanese adults who participated in a care prevention project called “Kayoi-no-ba”. All participants were divided into Internet and non-Internet user groups according to the classification of a previous study. We assessed LS (standing test, two-step test, and five-question Geriatric Locomotive Function Scale), frailty (through the Questionnaire for Medical Checkup of Old-Old), and sarcopenia (grip strength, normal walking speed, and skeletal muscle mass index) and made group comparisons between Internet users and non-users. Binomial logistic regression analyses were performed with Internet use as the independent variable and sarcopenia or LS as the dependent variables. The Internet and non-Internet user groups had 69 and 36 participants, respectively. The Internet user group comprised 65.7% of all participants, which was similar to that reported in a previous study of the same age group. Between-group comparisons showed significant differences in sarcopenia and LS items, whereas adjusted binomial logistic analysis showed a significant association between sarcopenia and Internet use. In summary, among LS, frailty, and sarcopenia, sarcopenia showed the highest association with Internet use. Older adults without sarcopenia having good physical functions, such as grip strength, walking speed, and skeletal muscle index, more likely used the Internet; while older adults with sarcopenia were less likely to use the Internet. This implied that Internet use may be associated with physical function.

## 1. Introduction

In Japan, community health nursing is an academic discipline aimed at improving people’s quality of life and contributing to the development of healthy and safe communities that support this quality of life. It is necessary to target individuals living in diverse settings and at various health levels, to take a continuous and comprehensive view of their lives, and to explore effective nursing care in collaboration with individuals and communities.

The aging rate in Japan is 29.0%, and a super-aging society has already arrived [1]. To improve people’s quality of life in a rapidly aging society, extending healthy life expectancy and preventing long-term care is increasingly crucial. Among these, locomotive syndrome (LS), a condition in which mobility is impaired due to musculoskeletal disorders associated with adverse outcomes, frailty (a condition in which vulnerability to various stresses increases as physiological reserves decrease with age) and sarcopenia (a decline in skeletal muscle mass, muscle strength, and physical function with aging) are of the utmost importance [2,3,4]. In 2022, the Japanese Medical Science Federation released the “Declaration of the Medical Society to Overcoming Frailty and Locomotive Syndrome” [5,6], which states that to overcome LS and frailty, health care providers must work together to contribute to the health and longevity of the population and to maintain the physical activity level of individuals. Additionally, sarcopenia is considered a core component of frailty [7], so there is a need to assess LS, frailty, and sarcopenia comprehensively.

In addition, social isolation among older adults and an increased number of people have been recognized as serious issues in Japan [1]. Social isolation is defined as little or no contact with family or community [8]. Older adults who are less likely to go out and interact with others are at increased risk of needing nursing care and death [8,9,10]. Preventive measures like nurturing social capital are pivotal in health outcomes. Social capital is a sociological construct widely used to capture the value of our social relationships and focuses on social connections, resources, and civic participation [11]. One of the ways to foster social capital is through Internet use. In today’s digital society, middle-aged and older individuals are increasingly using the Internet to obtain and disseminate information and for non-face-to-face interaction. Thus, the Internet may play a role in promoting social capital among middle-aged and older adults [11].

Therefore, this study was focused on the Internet use of community-dwelling older adults. Currently, 92.1%, 79.9%, and 51.5% of older Japanese adults in their 60s, 70s, and ≥80s, respectively, own a mobile device (at least one type of cell phone or smartphone) [12]. Internet use among older adults is increasing annually, with 86.8% for people in their 60s, 65.5% for those in their 70s, and 33.2% for those in their 80s or older [12]. The percentage of respondents in their 60s, 70s, and ≥80s has increased yearly. In our previous survey, approximately 90% (mean age 78.2 ± 8.0 years) owned at least one cell phone or smartphone in 2020, revealing an association between ownership status and disuse syndrome [13]. Therefore, the Internet might be a helpful tool that should be utilized to promote the extension of healthy life expectancy and care prevention.

The association between Internet use and older adults has been reported in many studies. Internet use has been associated with higher social capital [11], positive effects on mental health, such as happiness and depression [14,15,16], and a tendency towards reduced frailty [17,18]. Therefore, it is clear that Internet use has been associated with older adults’ physical and mental functions [11,14,15,16,17,18]. Although reports have demonstrated the association between Internet use and frailty, no prior studies have reported on the association between Internet use and LS or sarcopenia. Furthermore, no studies have simultaneously investigated the association between Internet use and the following three conditions: LS, frailty, and sarcopenia. We hypothesized that frailty is a concept that includes social aspects and may be more closely associated with Internet use compared to LS and sarcopenia [3]. In a previous study, we investigated the prevalence of electronic device ownership among community-dwelling older adults while commuting to “Kayoi-no-ba” [13]. As the next step, this study aimed to clarify the prevalence of LS, frailty, and sarcopenia among community-dwelling older adults who use the “Kayoi-no-ba” approach and their relationship with Internet use. This study provides a comprehensive view of the lives of older residents in the community, enabling the exploration of nursing practices that can be effectively implemented in collaboration with individuals and communities.

## 2. Materials and Methods

### 2.1. Study Design

This cross-sectional study was conducted between July 2022 and March 2023 in City A, Tochigi Prefecture, Japan. The study city fell into the suburban category as of 14 May 2024, according to the population density classification in a previous study [19]. All participants were given a verbal and written explanation of the study, and written informed consent was obtained. This study was approved by the Ethical Review Board of the International University of Health and Welfare (approval number: 18-Io-158-2) and was conducted following the guidelines of the Declaration of Helsinki.

### 2.2. Participants

**Figure 1:**
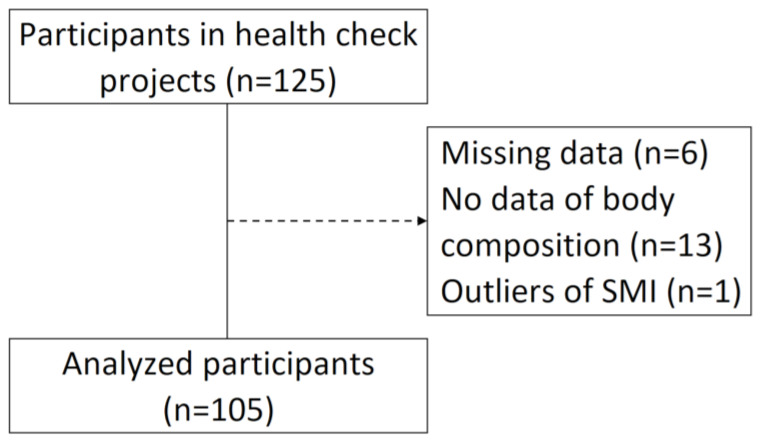
Flowchart of the participants. SMI: Skeletal muscle mass index.

Community-dwelling older adults (*n* = 125) who voluntarily participated in the health check project sponsored by the city were eligible for this study. The city office contacted the representative of the “Kayoi-no-ba” in each area, and the representative contacted the residents for potential participants [20]. A “Kayoi-no-ba” serves as a communal hub where older residents can engage in health promotion activities such as physical exercise and hobbies, facilitated by local volunteers, with overall support from the local government [20]. Participants with missing data, whose body compositions were not measured due to time constraints or refusal, or whose skeletal muscle indices (SMI) were outliers were excluded. In total, 105 older adults were included in the analysis (mean age: 79.1 ± 7.4 years). The flowchart of participant selection is shown in Figure 1.

### 2.3. Questions about Internet Use and Group Allocation

This study conducted face-to-face interviews using a questionnaire to assess Internet use based on the report by Satake et al. [21]. The question was, “Do you use the Internet including e-mail or other communicative applications with computer, smartphone, or tablet devices?”. Participants then chose one of three responses regarding their use in the past month: (i) “Yes, I use the Internet without help”, (ii) “Yes, I use the Internet with help”, or (iii) “No, I do not use the Internet”. On the basis of the same groupings as in a previous study, participants who responded to (i) or (ii) were placed in the Internet users group, whereas those who responded to (iii) were placed in the non-Internet users group [21].

### 2.4. Assessment of Locomotive Syndrome

The standing test, two-step test, and 5-question Geriatric Locomotive Function Scale (GLFS-5) were used (https://locomo-joa.jp/) (accessed on 14 May 2024). The standing test assessed whether the participant could stand up on one leg from a 40 cm-high stool and on both legs from 30 and 20 cm-high stools. Participants were considered to have passed the height test if they could stand up without leaning to gain momentum and hold the posture for 3 s. Participants took two steps forward at a maximum stride in the two-step test. The distance traveled during the two strides was measured (cm) and divided by the height of each individual (cm) [2]. The maximum value of two measurements was taken as the representative value. The GFLS-5 is a simplified version of the 25-question Geriatric Locomotive Function Scale, consisting of five items scoring from 0 to 20, indicating no disability to most severe disability [22]. The GFLS-5 consists of questions involving going up and down the stairs, walking briskly, walking distance without resting, carrying objects weighing approximately 2 kg, load-bearing tasks, and housework (Table 1) [22].

LS is determined using three tests: the standing test, two-step test, and 25-question Geriatric Locomotive Function Scale (GLFS-25). These three assessments have already been proven to be valid and reliable, and they have also been shown to be quantifiable assessment measures that can be used in a wide range of ages, from young to old [22,23,24]. In this study, owing to the city-sponsored health program, we used the GLFS-5, a simplified version of the GLFS-25 [22]. Participants were judged to have LS if they could not stand up from a 40 cm-high stool using either leg and/or scored <1.3 in the two-step test and/or ≥6 on the GFLS-5 [2,22]. In other words, if any one of the tests met the criteria, the participant was judged to have LS.

### 2.5. Assessment of Frailty

Frailty was assessed using the Questionnaire for Medical Checkup of Old-Old (QMCOO), which is a frailty assessment that was released in Japan in April 2020 [25]. The QMCOO consists of 15 questions in 10 domains, including health condition, mental health, eating behavior, oral function, body weight loss, physical function and falls, cognitive function, smoking, social participation, social support, and burden on older adults. The total score ranges from 0 to 15, with higher scores indicating poorer health. The QMCOO has validity with the J-CHS criteria and Kihon Checklist established as frailty assessments [26,27]. Additionally, the QMCOO is a questionnaire for older adults in their later stages of life; however, it has also been shown to determine frailty in older adults in their earlier stages of life and has predictive validity for the need for new care [28,29]. The Japanese version has been translated into English [25]. The present study defined frailty as a QMCOO score of ≥4 [28,30].

### 2.6. Assessment of Sarcopenia

Body weight, body mass index (BMI), fat mass, body fat percentage, and SMI were measured using a multi-frequency body composition analyzer (MC-780AN, TANITA, Tokyo, Japan). SMI was calculated by dividing the limb muscle mass by the height squared. Grip strength was measured using a Smedley-type grip dynamometer (T.K.K. 5401, Takei Kiki Kogyo Co., Ltd., Niigata, Japan), with the maximum value being the representative value. Walking speed was measured once at a normal speed on a 4 m or 5 m walking path. Sarcopenia was determined following the Asian Working Group for Sarcopenia 2019, and cutoff values for each assessment are as follows [31].

Grip strength: <28 kg in males, <18 kg in females
Normal walking speed: <1 m/s in males and females
SMI: <7.0 kg/m^2^ in males and <5.7 kg/m^2^ in females

Grip strength indicates muscle strength, normal walking speed indicates physical function, and SMI indicates skeletal muscle mass. Sarcopenia was defined as the loss of SMI, grip strength, and/or normal walking speed. Figure S1 (Supplementary Materials) shows the measurement situations of grip strength and body composition.

### 2.7. Statistical Analysis

Statistical analyses were performed using the unpaired *t*-test, Mann–Whitney U, Chi-square, and Fisher’s exact tests to compare the groups with and without Internet use. Age, height, weight, BMI, fat mass, body fat percentage, two-step value, grip strength, normal walking speed, and SMI were analyzed using an unpaired *t*-test. The total points of the GLFS-5 and QMCOO were analyzed using the Mann–Whitney U test. Gender, presence of LS, frailty, and sarcopenia were analyzed using the Chi-square or Fisher’s exact test.

The sarcopenia and LS items were significant in the between-group comparison of Internet use or not; therefore, binomial logistic regression was conducted to analyze the association of sarcopenia or LS with Internet use. In the analysis, the presence/absence of sarcopenia or LS was the dependent variable and Internet use was the independent variable. In Model I, there were no adjustment variables. Considering the sample size of this study, the only adjustment variables in Model II other than sex were age and BMI. In a logistic regression analysis, the number of participants in a small number of events should be at least about 10 times the number of input variables [32]. LS, frailty, and sarcopenia were used as adjustment variables in the multivariate analyses of many previous studies because of the influence of age, sex, and BMI [2,19,29,33,34,35]. Post hoc power analysis was performed on the binomial logistic regression analysis results. Statistical analyses were performed using IBM SPSS version 25 (IBM Japan, Tokyo, Japan) and G*Power version 3.1.9.2. The statistical significance level was set at *p* < 0.05.

## 3. Results

The distribution of participants answering the following question, “Do you use the Internet including e-mail or other communicative applications with computer, smartphone, or tablet devices?”, with one of the three possible answers was as follows: (i) “Yes, I use the Internet without help”: 59 participants (56.2%), (ii) “Yes, I use the Internet with help”: 10 participants (9.5%), and (iii) “No, I do not use the Internet”: 36 participants (34.3%).

**Figure 2:**
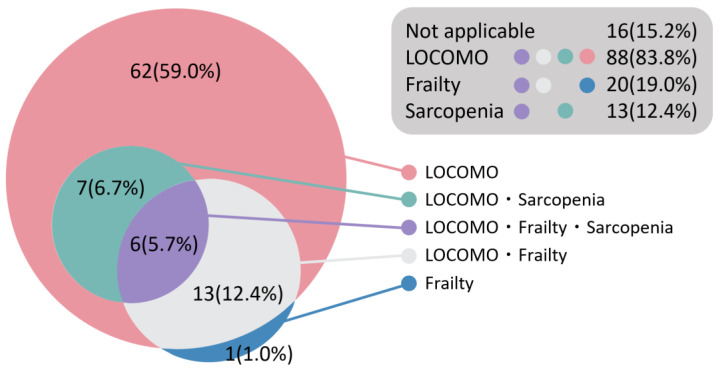
The prevalence of locomotive syndrome, frailty, and sarcopenia in this study. LOCOMO: Locomotive syndrome.

The prevalence of LS, frailty, and sarcopenia was 88 (83.8%), 20 (19.0%), and 13 (12.4%), respectively, whereas 16 participants (15.2%) did not present any of these (Figure 2). Notably, all participants with sarcopenia and 19 of the 20 participants with frailty also presented with LS. Of the 88 participants with LS, 26 (29.5%) had frailty and/or sarcopenia.

The comparison results between the groups are shown in Table 2 and Table 3. Of the basic attributes, the age (*p* < 0.001), height (*p* = 0.007), and body fat percentage (*p* = 0.024) showed significant differences. Compared with the Internet users, the non-Internet users were older, shorter, and had a higher body fat percentage. In terms of main outcomes, significant differences were found in LS (*p* = 0.006) and sarcopenia (*p* = 0.027). Moreover, significant differences were found in the following LS and sarcopenia assessment items: one leg 40 cm (*p* = 0.001), both legs 20 cm (*p* = 0.001), both legs 30 cm (*p* = 0.003), two-step value (*p* < 0.001), grip strength (*p* = 0.014), walking speed (*p* = 0.002), and SMI (*p* = 0.040). No significant differences were found in the presence of frailty, QMCOO (total score), or the QMCOO questionnaire items (Table 3). Table S1 (Supplementary Materials) shows a comparison of the number of items that met the criteria for LS between the Internet user and non-Internet user groups. The binomial logistic regression analysis results are presented in Table 4. Internet use was independently associated with sarcopenia in Model I (odds ratio = 3.657, β = 1.297, *p* = 0.035) and Model II (odds ratio = 4.431, β = 1.489, *p* = 0.031). The post-hoc statistical power was 0.86 for the non-adjusted Model I and 0.96 for the adjusted Model II. The Hosmer–Lemeshow test on the adjusted model showed a good fit with *p* ≥ 0.05. As for LS, the regression equation was significant in the non-adjusted Model I (odds ratio = 10.566, β = 2.358, *p* = 0.025) but not in Model II, which included the adjustment variables.

## 4. Discussion

Focusing on the use or non-use of the Internet, this study clarifies the prevalence of LS, frailty, and sarcopenia among community-dwelling older adults and the association of LS and sarcopenia with Internet use. The findings in relation to frailty and sarcopenia were unexpected. We demonstrated that Internet use was highly associated with sarcopenia, a decline in skeletal muscle mass, muscle strength, and physical function with aging.

The mean age of all participants in the present study was 79.1 ± 7.4 years, and 69 participants (65.7%) were Internet users, which was similar to a previous report [12]. The prevalence of LS, frailty, and sarcopenia is reported to be 81.0%, 19.6%, and 9.9%, respectively, in community-dwelling older adults in Japan [30,36,37]. The prevalence rates in this study are almost comparable, being 83.8%, 19.0%, and 12.4%, respectively [30,36,37]. On the other hand, in this study, the rate of participants with LS combined with frailty and/or sarcopenia (29.5%) was lower than that reported in a previous study on community-dwelling older adults (41.1%) [38]. Therefore, this study’s participants may be a relatively healthier population than that of previous studies.

Regarding the association of LS, frailty, and sarcopenia with Internet use, only sarcopenia was associated with Internet use in the age-adjusted multivariate analysis. Regarding LS, the regression equation was insignificant due to the high prevalence and strong effect of age. For frailty, the QMCOO is structured with questions regarding oral frailty (Nos. 4 and 5), physical frailty (Nos. 6–9), cognitive frailty (Nos. 10–11), social frailty (Nos. 13–15), and evaluations of physical, cognitive, and social frailty (Nos. 1–3). The fact that no significant differences in these questions were found between Internet users and non-Internet user groups contradicts our hypothesis. This is still a debate; however, the fact that the engaged participants took part in the health check program voluntarily may have been why social capital and social aspects of frailty were not extracted as relevant factors.

The study also found that the Internet user group was younger and that there were differences between the Internet user and non-user groups in all measured physical parameters, particularly for grip strength, walking speed, SMI, standing test results, and two-step value. In a previous study, not owning a smartphone or a computer was associated with inactivity in similar individuals participating in a health check program [13]. Notably, Internet use has been shown to improve quality of life by improving access to medical services, influencing individuals to adhere to a healthy lifestyle and support social participation, providing a means of accessing information for self-management of diseases, and having a high likelihood of being healthy [17,39,40,41]. It has been reported that Japanese adults aged ≥ 65 years often search the Internet for hospitals and other medical facilities and disease characteristics, such as names of diseases, symptoms, and treatments, to obtain useful information about their health and medical conditions [1,42]. Furthermore, Internet use was associated with a higher grip strength, which was true even after adjusting for age, smoking, physical activity, chronic diseases, sitting time, and other factors, supporting our study’s results [43]. Furthermore, some reports have suggested that ICT use is associated with quality of life and health literacy [44,45,46]. Integrating and interpreting our results with the findings of these previous studies, these findings suggest that healthy people are more likely to own information and communication devices, such as smartphones, and to use the Internet to maintain their health and Internet use is associated with good performance, such as sarcopenia and LS. Conversely, the findings suggest that sarcopenic individuals with low muscle strength and physical function are less likely to use the Internet.

This study has some limitations. First, since this is a cross-sectional study, longitudinal studies are needed to elucidate the cause–effect relations (internet use and physical function). Second, owing to the limited time, data on the frequency of Internet use, activities of daily living, cognitive function, and socioeconomic status were not collected. In addition, only three adjustment variables for multivariate analysis could be entered due to the sample size. Third, this study was also conducted in a single city of suburban category and only among participants of a health check project. Additionally, there may be selection bias because older adults who voluntarily participate in health check programs were used as the target group. Fourth, we used the GLFS-5, a simplified version of the GLFS-25. However, the prevalence of LS, frailty, and sarcopenia was clarified based on formal actual measurements, and their relationship with Internet use was elucidated. This study’s results will contribute to supporting older adults living in communities that use “Kayoi-no-ba” for health check projects, such as part of a population strategy approach to promote and raise awareness of health.

## 5. Conclusions

This study investigated the association of Internet use with LS, frailty, and sarcopenia among older adults living in the community who participated in a “Kayoi-no-ba” for a health check project. The results showed that participants without sarcopenia and those with good grip strength, walking speed, and SMI were more likely to use the Internet; while older participants with sarcopenia were less likely to use the Internet. The presence or absence of Internet use may be associated with sarcopenia-related factors, such as physical function, muscle strength, and skeletal muscle mass. Further longitudinal investigations are required to explore the causal relationships associated with aging symptoms and Internet use.

## Figures and Tables

**Table 1:** The 5-question Geriatric Locomotive Function Scale.

1. To what extent has it been difficult to go up and down the stairs? Not difficult, Mildly difficult, Moderately difficult, Considerably difficult, Extremely difficult 2. To what extent has it been difficult to walk briskly? Not difficult, Mildly difficult, Moderately difficult, Considerably difficult, Extremely difficult 3. How far can you keep walking without rest? (please select the closest answer) More than 2–3 km, approximately 1 km, approximately 300 m, approximately 100 m, approximately 10 m 4. To what extent has it been difficult to carry objects weighing approximately 2 kg (2 standard milk bottles or 2 PET bottles, each containing 1 L)? Not difficult, Mildly difficult, Moderately difficult, Considerably difficult, Extremely difficult 5. To what extent have load-bearing tasks and housework (cleaning the yard, carrying heavy bedding, etc.) been difficult? Not difficult, Mildly difficult, Moderately difficult, Considerably difficult, Extremely difficult

**Table 2:** Comparison between Internet user and non-Internet user groups.

	Internet User (*n* = 69)	Non-Internet User (*n* = 36)	*p* Value
Age (years)	76.7 ± 7.0	83.5 ± 6.1	<0.001 *
Sex (women)	52 (75.4)	31 (86.1)	0.199
Height (cm)	153.1 ± 7.3	148.9 ± 7.7	0.007 *
Weight (kg)	55.1 ± 10.0	54.5 ± 10.7	0.743
BMI (kg/m^2^)	23.5 ± 3.5	24.5 ± 3.9	0.174
Fat mass (kg)	17.6 ± 6.4	19.5 ± 7.2	0.169
Body Fat Percentage (%)	31.3 ± 8.1	35.1 ± 8.0	0.024 *
Presence of LS	53 (76.8)	35 (97.2)	0.006 *
One leg 40 cm (fail)	46 (66.7)	34 (94.4)	0.001 *
Both legs 20 cm (fail)	5 (7.2)	12 (33.3)	0.001 *
Both legs 30 cm (fail)	2 (2.9)	8 (22.2)	0.003 *
2-step value (cm/height)	1.2 ± 0.2	1.0 ± 0.2	<0.001 *
GLFS-5 (≥6 points)	16 (23.2)	10 (27.8)	0.605
GLFS-5 (total points)	2.0 (0.0–5.0)	4.0 (1.25–6.0)	0.055
Presence of frailty (≥4 points)	13 (18.8)	7 (19.4)	0.940
QMCOO (total points)	2.0 (1.0–3.0)	2.0 (1.0–3.0)	0.730
Presence of sarcopenia	5 (7.2)	8 (22.2)	0.027 *
Grip strength (kg)	24.8 ± 7.4	21.2 ± 6.1	0.014 *
Normal walking speed (m/s)	1.3 ± 0.2	1.2 ± 0.2	0.002 *
SMI (kg/m^2^)	6.6 ± 0.8	6.3 ± 0.8	0.040 *

* *p* < 0.05. Data are presented as mean ± standard deviation (SD), number (%), or median (25th percentile–75th percentile). BMI: body mass index, GLFS-5: five-question Geriatric Locomotive Function Scale, LS: locomotive syndrome. QMCOO: Questionnaire for Medical Checkup of Old-Old, SMI: skeletal muscle mass index.

**Table 3:** Comparisons of sub-items of the Questionnaire for Medical Checkup of Old-Old between the Internet user and non-Internet user groups.

Domain	No.	Item	Answer		Internet User (*n* = 69)	Non-Internet User (*n* = 36)	*p* Value
Health condition	1	How is your health condition?	Excellent/Good/Fair	0	65 (94.2)	34 (94.4)	1.000
			Poor/Very poor	1	4 (5.8)	2 (5.6)	
Mental health	2	Are you satisfied with your daily life?	Satisfied/ Moderately satisfied	0	64 (92.8)	36 (100.0)	0.162
			Moderately dissatisfied/Dissatisfied	1	5 (7.2)	0 (0.0)	
Eating behavior	3	Do you eat three times a day?	Yes	0	67 (97.1)	35 (97.2)	1.000
			No	1	2 (2.9)	1 (2.8)	
Oral function	4	Do you have any difficulties eating tough foods compared to 6 months ago?	No	0	51 (73.9)	25 (69.4)	0.627
			Yes	1	18 (26.1)	11 (30.6)	
	5	Have you choked on your tea or soup recently?	No	0	53 (76.8)	29 (80.6)	0.660
			Yes	1	16 (23.2)	7 (19.4)	
Body weight loss	6	Have you lost 2 kg or more in the past 6 months?	No	0	61 (88.4)	33 (91.7)	0.745
			Yes	1	8 (11.6)	3 (8.3)	
Physical function and falls	7	Do you think you walk slower than before?	No	0	38 (55.1)	15 (41.7)	0.192
			Yes	1	31 (44.9)	21 (58.3)	
	8	Have you experienced a fall in the past year?	No	0	59 (85.5)	32 (88.9)	0.767
			Yes	1	10 (14.5)	4 (11.1)	
	9	Do you go for a walk for your health at least once a week?	Yes	0	49 (71.0)	24 (66.7)	0.646
			No	1	20 (29.0)	12 (33.3)	
Cognitive function	10	Do your family or friends point out your memory loss? (e.g., “You ask the same question over and over again”.)	No	0	56 (81.2)	28 (77.8)	0.681
			Yes	1	13 (18.8)	8 (22.2)	
	11	Do you find yourself not knowing today’s date?	No	0	55 (79.7)	27 (75.0)	0.580
			Yes	1	14 (20.3)	9 (25.0)	
Smoking	12	Do you smoke?	No	0	58 (84.1)	31 (86.1)	0.781
			I quit/Yes	1	11 (15.9)	5 (13.9)	
Social participation	13	Do you go out at least once a week?	Yes	0	67 (97.1)	34 (94.4)	0.605
			No	1	2 (2.9)	2 (5.6)	
	14	Do you keep regular communication with your family and friends?	Yes	0	69 (100.0)	36 (100.0)	-
			No	1	0 (0.0)	0 (0.0)	
Social support	15	When you are not feeling well, do you have anyone you can talk with?	Yes	0	67 (97.1)	36 (100.0)	0.545
			No	1	2 (2.9)	0 (0.0)	

Data are presented as numbers (%). Statistical analysis was performed using the chi-square and Fisher’s exact tests.

**Table 4:** Association between sarcopenia and Internet use by binomial logistic regression analysis.

	β	Odds Ratio	95% CI	*p* Value
Model I	1.297	3.657	1.099–12.171	0.035 *
Model II	1.489	4.431	1.142–17.185	0.031 *

* *p* < 0.05. Dependent variable: presence of sarcopenia = 1, absence of sarcopenia = 0. Independent variable: non-Internet user = 1, Internet user = 0. Model I: Non-adjusted. Model II: Age, sex, and body mass index as adjustment variables. CI: confidence interval.

## Data Availability

Dataset available on request from the authors.

## Supplementary Materials

The following supporting information can be downloaded at: https://www.mdpi.com/article/10.3390/nursrep14020105/s1. Figure S1: Measurement situation of grip strength and body composition; Table S1: Comparison of the number of items that met the criteria for locomotive syndrome between Internet user and non-Internet user groups.

## Public Involvement Statement

There was no public involvement in any aspect of this research.

## Guidelines and Standards Statement

This manuscript was drafted against the Strengthening the Reporting of Observational Studies in Epidemiology (STROBE) guidelines for cross-sectional study.
