# Supplementary material for: Association between Internet Use and Locomotive Syndrome, Frailty, and Sarcopenia among Community-Dwelling Older Japanese Adults
Source: Nurs Rep. 2024 May 31;14(2):1402–13. doi: 10.3390/nursrep14020105 (PMC11207118; doi:10.3390/nursrep14020105)
Supplement: Supplementary file 1 [file nursrep-14-00105-s001.zip › nursrep-2895909-supplementary.pdf]

Figure S1. Measurement situation of grip strength and body composition.

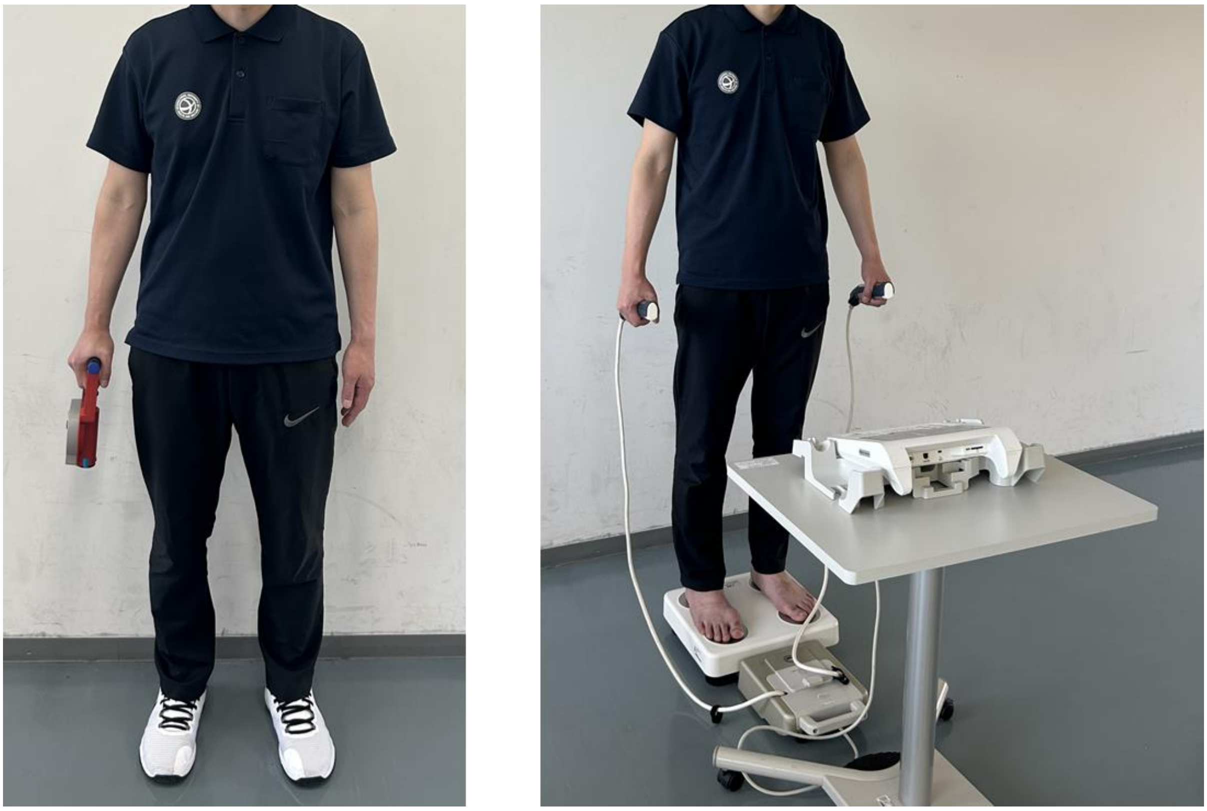

The left figure shows the measurement of grip strength, and the right figure shows the measurement of body composition.

Table S1. Comparison of the number of items that met the criteria for locomotive syndrome between Internet user and non-Internet user groups

	**Internet user (*n*=69)**	**Non-Internet user (*n*=36)**	***P*-value**
Number of LS applicable items			
0	16 (94.1)	1 (5.9)	0.001*
1	19 (86.4)	3 (13.6)	
2	22 (50.0)	22 (50.0)	
3	12 (54.5)	10 (45.5)	

*p<0.05.

Data are presented as number (%).

Number of LS applicable items mean the total number of items that met the criteria of the standing test, two-step test, and 5-question Geriatric Locomotive Function Scale.

LS: locomotive syndrome.
